# Patient-reported outcome measures in the interaction between patient and clinician – a multi-perspective qualitative study

**DOI:** 10.1186/s41687-019-0170-x

**Published:** 2020-01-09

**Authors:** Caroline Trillingsgaard Mejdahl, Liv Marit Valen Schougaard, Niels Henrik Hjollund, Erik Riiskjær, Kirsten Lomborg

**Affiliations:** 1 AmbuFlex/WestChronic, Occupational Medicine, University Research Clinic, Aarhus University, Herning, Gl Landevej 61, DK-7400 Herning, Denmark; 2 The Research Centre for Patient Involvement, Aarhus University & the Central Denmark Region, Palle Juul-Jensens Boulevard 99, DK-8200 Aarhus, Denmark; 3 Department of Clinical Epidemiology, Aarhus University Hospital, Aarhus, Olof Palmes Allé 43-45, DK-8200 Aarhus, Denmark; 4 DEFACTUM, Social & Health Services and Labour Market, Olof Palmes Allé 15, DK-8200 Aarhus, Central Denmark Region Denmark; 5 Department of Clinical Medicine, Aarhus University, Palle Juul-Jensens Boulevard 82, DK-8200 Aarhus, Denmark

**Keywords:** Patient-reported outcome (PRO) measures, Outpatient follow-up, Qualitative research, Interpretive description, Patient-clinician interaction, Communication

## Abstract

**Background:**

This article addresses patient-reported outcome (PRO)-based follow-up used as a substitute for regularly scheduled follow-ups. In PRO-based follow-up, patients’ PRO data filled in by the patients at home are used by clinicians as a decision aid to identify those who need clinical attention based on an automated PRO algorithm, clinical attention being either a phone call or a physical consultation. A physical consultation in the outpatient clinic prompted by the patient’s PRO is termed a “PRO consultation.”

In this multi-perspective qualitative study, we explored the influence of patients’ self-reported data on patient-clinician interaction during PRO consultations in epilepsy outpatient clinics. Interpretive description was the methodological approach, applying data from participant observations, informal interviews with clinicians, and semi-structured interviews with clinicians and patients.

**Results:**

We found that application and deliberate use of patients’ PRO measures can affect patient-clinician interaction, promoting patient involvement in terms of improved communication and increased patient activation. These findings reflect the general patterns that have been reported in the literature. In addition, we found that PRO measures also may induce unmet expectations among some patients that can have a negative effect on patients’ experiences of the interaction and their follow-up experience in general. We extracted two thematic patterns that represent PRO measures’ potential for patient involvement in the patient-clinician interaction. The first pattern represents enablers, and the second pattern represents barriers for PRO measures to affect patient involvement.

**Conclusions:**

Applying PRO measures in clinical practice does not automatically enhance the patient-clinician interaction. To strengthen the benefits of PRO measures, the following supplementary clinical initiatives are suggested: summarizing and reporting the PRO measures back to the patient, considering carefully which PRO measures to include, training clinicians and assuring that the patients’ introduction to PRO-based follow-up clarifies expectations.

## Introduction

A patient-reported outcome (PRO) measure is defined as “*a measurement based on a report that comes directly from the patient about the status of a patient’s health condition without amendment or interpretation of the patient’s response by a clinician or anyone else.*” [1]. Thus, PRO measures seek to ascertain patients’ views of their symptoms, functional status, well-being, and health-related quality of life [2–4]. At the individual level in clinical practice, PRO measures can be used to screen for relevant symptoms, monitor progress over time, and support clinical decisions [5, 6]. PRO measures may also be used to further strengthen patients’ active participation in their own care [7–10] and are believed to facilitate patient involvement considerably [6, 11–13]. PRO measures are expected to improve patient-clinician communication [14, 15] in general and patient-centered communication in particular [16–20].

In outpatient settings, PRO measures can be seen as a precursor of the upcoming consultation, legitimizing its relevance. Although PRO measures hold much potential, research shows that applying PRO measures in clinical practice does not automatically promote patient involvement and improve communication [21, 22]. A number of barriers impeding the improvement of healthcare quality through PRO measures have been identified. They may be divided into practical, attitudinal, and methodological barriers [8, 14]. Thus, a series of conditions and mechanisms may influence the use of PRO measures in patient-clinician interaction.

In the study we explored the influence of PRO measures on patient-clinician interaction in epilepsy outpatient clinics.

## Methods

### Interpretive description

Interpretive description (ID) is an applied, inductive research strategy emphasizing the significance of performing research to improve clinical practice [23]. ID differs from other methodologies by drawing on elements derived from phenomenology, grounded theory, and ethnography while refraining from formalizing specific techniques and procedures as ultimate standards and goals of research [23]. As required for ID studies, our data generation and analysis were iterative, letting the preliminary analysis guide the subsequent data collection. Thus, we compared, reflected upon, and explored data elements throughout the process and across empirical data sources.

### Setting

Our starting point was outpatient follow-up for epilepsy, where an already implemented PRO solution, AmbuFlex, provided the opportunity to explore how PRO measures influence patient-clinician interaction. AmbuFlex is a generic web-based PRO system that supports demand-driven outpatient follow-up [11, 24]. The overall aims of AmbuFlex are to improve quality of care, increase patient-centered care, and relocate health service resources. Regularly scheduled follow-up is replaced by regular questionnaires filled in by the patients at home. Clinicians use the patients’ self-reported PRO data as a decision aid to identify those who need attention [25, 26]. In 2012, AmbuFlex was implemented in three epilepsy outpatient clinics in Central Denmark Region. Prior to implementing AmbuFlex, follow-up for patients with epilepsy was managed by regular pre-scheduled visits, typical every 6th or 12th month.

AmbuFlex was initially implemented at Aarhus University Hospital on request from clinicians in the epilepsy outpatient clinic. PRO-based follow-up was developed in close collaboration between clinicians and an AmbuFlex team. Patients were only involved in the development of the questionnaire. Subsequently, AmbuFlex/epilepsy was implemented in the epilepsy outpatient clinics at Holstebro Regional Hospital and Viborg Regional Hospital.

The questionnaire has been regularly revised and changed according to mutual agreement amongst clinicians from the three epilepsy clinics. Initially the nurses received technical training in using the PRO system; however, there was no formal follow-up or ongoing training of the clinicians. Physicians receive no training in the PRO system.

In September 2019, approximately 3000 epilepsy outpatients used AmbuFlex in Central Denmark Region, which is approximately 50% of epilepsy patients in the region. Clinicians refer patients to AmbuFlex based on an assessment of their health status and ability to fill in the PRO questionnaires. Thus, AmbuFlex is the standard procedure in only half of the epilepsy patients because the clinicians assessed that a large proportion of patients would not be able to use or gain benefit from this type of follow-up. In addition, patients have the possibility to decline participation in PRO-based follow-up.

The questionnaire encompasses information specific to aspects of daily life with epilepsy, e.g. seizures, side effects, well-being, general health, and social problems (Additional file 1). PRO responses are automatically given a specific algorithm: a “green”, “yellow”, or “red” status. “Red” indicates that the patient needs/wishes contact, “green” that there is no current need for attention, while “yellow” indicates that the patient may need contact, but a clinician has to decide whether this is needed. The patients can always request contact, either a phone call or a consultation in the outpatient clinic. This will automatically overrule any decision that no visit is needed [26]. We termed a physical consultation in the outpatient clinic prompted by the patient’s PRO a “PRO consultation”.

### Sampling and data collection

Data were obtained from the following sources: (1) Field studies comprising participant observations in PRO consultations and subsequent informal interviews with clinicians, (2) Individual, semi-structured interviews with patients, (3) Individual, semi-structured interviews with clinicians. Data were collected by the first author. We combined observations of consultations and patients and clinicians’ reflections to achieve a nuanced understanding of the mechanism of action related to the patient-clinician interaction during PRO consultations.

#### Field studies (participant observations and informal interviews)

The studies (conducted from September 2016 to June 2017) centered on patients who had either requested a consultation via the PRO questionnaire or whose answers in the questionnaire had prompted the clinicians to schedule a PRO consultation. Nurses in the outpatient clinics contacted 32 potential participants prior to their consultation and secured their consent to participate. All 32 participants agreed to participate; however, due to circumstances in the outpatient clinics, 9 of the planed participant observations were cancelled on the day the consultation was scheduled. Thus, only 23 participant observation sessions were conducted (Table 1). Field notes were taken, either during participant observations or immediately afterwards, including notes from informal interviews with clinicians. To promote consistency in the observations and field notes, a semi-structured observations guide was used. The observation guide entailed (a) how the patient’s PRO data were applied in the consultation, (b) how PRO data were articulated in the patient-clinician interaction, (c) how PRO measures affected communication between the two, and (d) further actions or initiatives prompted by the patient’s PRO measures. Ten participant observations were followed up by an immediate informal interview with the clinician in question to explore his or her impression of the encounter with the patient and allow the clinician to comment on aspects or actions observed during the consultation.

**Table 1:** Observations in PRO consultations

		*N* = 23 (%)
Gender (patient)	Female	12 (52)
	Male	11 (48)
Age (patients)	20–35	5 (22)
	36–50	5 (22)
	51–65	9 (39)
	> 65	4 (17)
Outpatient clinic	Aarhus	21 (91)
	Holstebro	2 (9)
Profession of clinician in charge of the consultation	Physician	21 (91)
	Nurse	2 (9)
Consultation prompted by	Patient’s request	11 (48)
	Clinical assessment of PRO questionnaire	12 (52)

#### Individual patient interviews

Observations of consultations were followed up by an in-depth individual interview with the specific patient within 1–4 weeks after the PRO consultation. Twelve patients were invited and 8 agreed to participate in these post-observation interviews. In these interviews, specific situations observed in the PRO consultation were discussed, and the patient was asked to share experiences from the specific PRO consultation and about PRO consultations in general. In addition, four individual interviews with patients who had been referred to PRO-based follow-up were conducted. All these patients had experiences from participation in one or more PRO consultations. The 12 patient interviews were conducted in the patients’ homes, except for one interview which took place at the hospital on request of the patient (Table 2).

**Table 2:** Patient participant profile, individual interviews

		*N* = 12 (%)
Gender	Female	6 (50)
	Male	6 (50)
Age (years)	20–35	4 (33)
	36–50	1 (8)
	51–65	5 (42)
	> 65	2 (17)
Outpatient clinic	Holstebro	1 (8)
	Aarhus	11 (92)
Duration of epilepsy (years)	< 5	1 (8)
	6–45	10 (84)
	> 45	1 (8)
Cohabitation	Living with a partner	11 (92)
	Living alone	1 (8)
Occupational status	Working	7 (58)
	Not working	5 (42)

#### Individual clinician interviews

Observation of consultations was followed up by in-depth individual interviews with the 6 clinicians in charge of the observed consultations (2 nurses and 4 physicians). Specific situations observed were discussed and the clinician was invited to share their experiences with the specific PRO consultations and with PRO consultations in general. Additionally, 7 individual interviews with clinicians working with AmbuFlex/epilepsy were conducted. These clinicians were invited to share their experiences with the influence of PRO-based follow-up on patient-clinician interaction (Table 3).

**Table 3:** Clinician participant profile, individual interviews

		No. 13 (%)
Profession	Nurse	8 (62)
	Physician	5 (38)
Gender	Female	10 (77)
	Male	3 (23)
Hospital	Holstebro	2 (15)
	Viborg	4 (31)
	Aarhus	7 (54)
Experience with AmbuFlex (months)	6–12	2 (15)
	13–24	1 (8)
	25–36	3 (23)
	> 36	7 (54)

### Data analysis

All individual interviews were audio-recorded with the participants’ permission and transcribed verbatim by the first author. The transcripts were immediately anonymized, and each participant was given a code number. Immediately after the PRO consultation, field notes were typed up. Data management was facilitated by the qualitative software program NVivo™ [27].

The first and last author collaborated on the analysis, supported by discussions with co-authors. Initially, we approached the data by reading all of the transcripts and field notes to develop a sense of the whole beyond our immediate impression of the material and across data sources. Thereafter, we arranged the data in terms of patterns that seemed to reflect similar properties. Based on these preliminary thematic patterns, we developed a matrix providing an overview of the possible coherence between the thematic patterns and the perspectives in the various empirical data sources. The development of this matrix was an iterative process in which the patterns were gathered and disassembled several times. Finally, we conceptualized the findings by extracting two themes that represented the influence of PRO measures on the interaction, based on a comprehensive analysis across the various empirical data sources.

### Ethical considerations

The Danish Data Protection Agency (Identification no. 2015-41-4119) approved the study. In keeping with the ethical principles for medical research involving patients stated by the World Medical Association Declaration of Helsinki [28], informed written consent was obtained and national requirements for health science research were followed.

## Results

In the interpretive description, we extracted two thematic patterns that represent PRO measures’ potential for patient involvement in the patient-clinician interaction. The first pattern represents enablers, and second pattern represents barriers for PRO measures to affect patient involvement. Each thematic pattern contained three sub-themes.

**Fig. 1:**
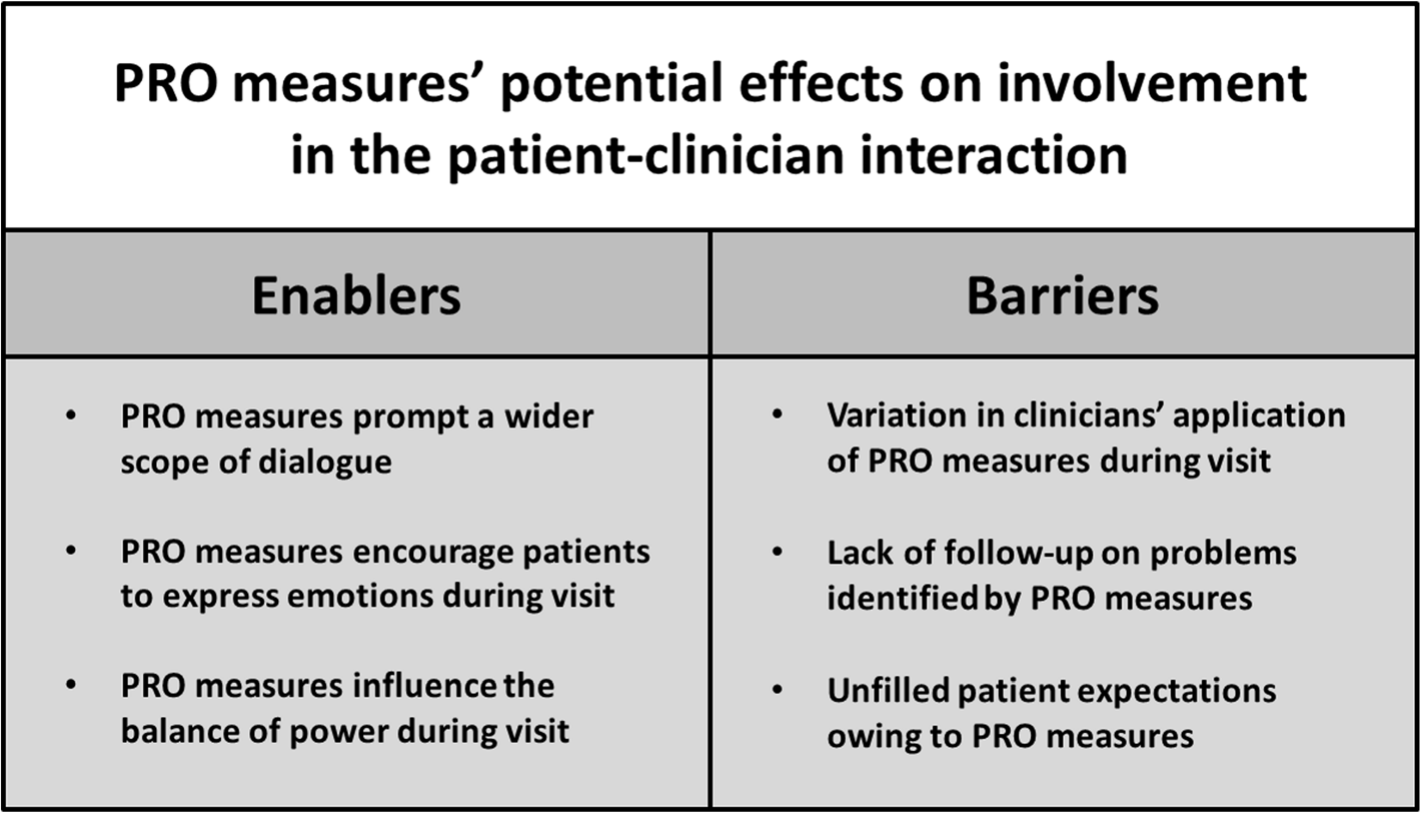
PRO measures’ potential effects on involvement in the patient-clinician interaction

The thematic patterns and sub-themes were elucidated by the three different perspectives and illustrated by participant quotes and excerpts from field notes. Figure 1 illustrates the findings.

### Enablers

#### PRO measures prompt a wider scope of dialogue

In several of the PRO consultations, the patients’ PRO measures seemed to guide the focus of the consultation. It was observed that dialogues about psychosocial problems took up much of the time in these consultations. The areas of concern were broad and touched on various issues in relation to the patient’s everyday life, e.g. grandchildren, problems in relation to work, problems with appearance, problems with school attendance, marital problems, hobbies, problems with sexuality, diet, and exercise.

Patients emphasized that clinicians seemed to be better prepared for the consultation because of their PRO data. Thus, patients found that their PRO response added focus to the consultation and prompted a relevant scope of the dialogue. Patients requested that their problematic PRO responses were the center of attention in the PRO consultation. When the dialogue was based on the patient’s PRO response, patients perceived that the consultation was more relevant and personal. Conversely, patients found that it was a waste of time and annoying if the clinician asked questions in relation to PRO measures that the patient had noted were not a problem.

Clinicians found that the patient’s PRO data changed the scope of the consultation. Previously, the focus was narrower and restricted to seizures and medical treatment. Now, the scope of the dialogue was wider and covered many more aspects of the patient’s everyday life with epilepsy. Addressing this issue, a physician said:

*“There is plenty of contents in the consultation. It is not just a few lines of text and “the patient continues taking his medicine as usual”, I mean. You have explained it to them, and you have discussed it, and this or that issue may be viewed in this or that way, and that may not be a side-effect. Perhaps it is this stuff that the patient must cope with. In other words, they become very, well, heavy.”*

Nurses emphasized that the patient’s PRO response provided structure to the PRO consultation, as the response was used as a playbook for the patient dialogue. Thus, they found that the consultations had become more structured and focused on the primary problems.

#### PRO measures encourage patients to express emotions during visit

The patient’s PRO response was often used to initiate a dialogue concerning sensitive issues. In most cases, these dialogues were initiated by the clinician, who would say, e.g.; *“I can see in your response that you have noted some sadness. Is this a new issue for you? Maybe you can tell me what you think is the reason for this?”* Thus, clinicians used the patient’s PRO response to encourage the patient to participate in the consultation. It was observed that dialogues based on PRO responses prompted patients to express emotions and reveal fears and worries.

Patients experienced PRO measures as a means to legitimize dialogue on psychosocial problems. They found that PRO measures made it possible to initiate discussions concerning issues beyond the medical treatment of their epilepsy or the results of their blood tests. A 68-year-old man describes how PRO measures changed the conversation with the physician:

*“But that is how it [the conversation] ended this time, I mean about how one has become the relative in one or other situation. It led to a totally different conversation; it became a completely different conversation.”*

Furthermore, clinicians found that the PRO measures relating to psychosocial problems made it easier for them to raise sensitive topics, and PRO measures supported them in asking the patient about personal aspects related to living with epilepsy.

#### PRO measures influence the balance of power during visit

During consultations in which the PRO measures were applied, changes in the patient-clinician interaction were observed compared to the consultations without application of PRO measures. The changes observed were increased speaking time for the patient and a changed seating arrangement during the consultation. When PRO measures were applied, the physician would often move a bit away from the computer screen and turn the screen toward the patient, allowing the patient to see the response. In some cases, the clinicians brought out specific answer components from the patient’s response, e.g. saying: “as you can see, this question has suddenly turned red compared with your previous responses”. Often it was the clinician who brought up the PRO response. Only in a single case did the patient bring up the PRO response herself, as she wanted to discuss the dizziness she experienced and which she had become aware of when filling in the PRO questionnaire.

Clinicians emphasized that, overall, PRO measures had altered the character of the relation between them and their patients. Physicians emphasized that PRO-based follow-up made the patients more active during consultations. They experienced that the patients tended to take more initiatives to discuss a wider range of issues; owing to the PRO questionnaires, the patients asked more questions and seemed more curious about their epilepsy. Thus, physicians found that PRO-based follow-up allowed patients to voice their concerns, and this shift allowed patients – at least in part – to take control over the PRO consultation. Additionally, clinicians emphasized that PRO measures supported patients in offering their opinions and in sharing their beliefs about their health status. As one physician put it:

*“In the case of Ambuflex conversations, then it’s often different, and it actually often develops differently, it is not just about epilepsy (…) Sex life and all the questions at the Ambuflex are read carefully, and then, as a human being, you start thinking: what’s that got to do with my condition, what’s it got to do with my medication? So, you can actually say that the patient is involved more. The patient conducts the conversation; they’re the ones asking me the questions, not the other way around (…). They guide the conversation; it’s fair to say that they become more involved, and that is clear to me.”*

Hereby, clinicians experienced a shift in the balance of power in their interaction with patients owing to PRO-based follow-up.

However, the analysis of the patient interviews gives reason to consider whether this shift of power in the interaction was recognized primarily by clinicians. Despite being invited to contribute with their assessments and perspectives, patients emphasized that it was still the clinicians who decided which topics to discuss. In addition, patient emphasized that the clinicians were often genuinely interested only in problems relating to seizures or medicine. As a 68-year-old woman said: *“ Well, they don’t care about it* (psychosocial problems) *at all. The only thing they react to is the medicine stuff.”*

### Barriers

#### Variation in clinicians’ application of PRO measures during visit

The degree to which PRO measures were applied varied greatly. In 15 out of the 23 observed consultations (65%), the clinician opened the PRO questionnaire on the computer screen. The degree to which PRO measures were articulated in the consultations also varied. In most of the consultations during which the clinician opened the PRO response on the computer screen, the patient’s response was articulated by the clinician. However, in some cases, the response was articulated neither by the clinician nor by the patient even though it was opened on the computer screen. Patients emphasized that in their experiences there was considerable variation in the practical application of their PRO responses during the consultation. In some cases, patients were in doubt whether their PRO response had been commented on during the consultation or not. In addition, a few patients were unsure whether they were allowed to see their response. A 28-year-old woman said: *“Yes, I could see the screen; I’m not sure you’re supposed to?”*

Even though all the consultations were prompted by the patient’s recent PRO response, physicians emphasized that they did not always find it necessary to refer to the specific questionnaire in their meeting with the patient. At times, the physician would consult the specific PRO response prior to the consultation, and if the clinician assessed that there was nothing in the PRO response that needed attention, the PRO response was not commented on. In other instances, the physician was unaware that the consultation was prompted by a PRO questionnaire.

#### Lack of follow-up on problems identified by PRO measures

Observations revealed that the dialogues about PRO measures only seldom triggered further initiatives or actions. In a few PRO consultations, additional actions taken by the clinician following discussion of PRO data were observed, e.g. a physician sent a note to a patient’s general practitioner prompted by a dialogue about the patient’s PRO data that had revealed that the patient was not doing so well and that he probably suffered from anxiety and depression. Often if the issue of psychosocial problems was raised, the clinician and the patient would agree that the problems were not directly related to epilepsy or the medical treatment of epilepsy. In other cases, the clinician concluded that the problem had no direct relation to the patient’s medical treatment or that the issue would not be solved or improved by a change in medication.

Patients experienced that only problems relating to seizures or medicine prompted actions from the clinician. They emphasized that different PRO measures ranked differently when it came to actions taken in the outpatient clinic. Some patients became frustrated when clinicians addressed only these problems. They emphasized that talking about or just recording their problems was insufficient. A 57-year-old-man said:

*“It would be far better for the patients and also far cheaper for society in the long term if you kind of acted on the things you enquire about. Because we’re not just joking; the questions we ask are not random. These are things that we know may be problematic for epilepsy patients. And it should be said that if the things pointed out are real problems, then we need to do something about them. If, for instance, I had written that I had suffered five generalized epilepsy seizures and that I was having tremors constantly, and if I felt that my medication was insufficient, then someone would have reacted immediately! Because that’s an expertise they have at the department. But the things that they don’t know how to solve at the department, but which may affect other things, these things they don’t touch.”*

Clinicians acknowledged that some of the problems brought up by the patient’s PRO data were out of their area of expertise and beyond the scope of the consultation. Clinicians typically emphasized that all they could offer the patients in these situations was to listen to them, and several clinicians thought that just listening to the patients’ problems was perceived as a positive element.

#### Unfilled patient expectations owing to PRO measures

In a couple of instances, signs of unmet patient expectations were observed. In several situations, patients seemed disappointed when the clinician dismissed taking further actions on problems noted in the PRO questionnaire. Excerpt from field notes:

*The physician says that if the patient needs a workup for her headache and potential high blood pressure, it needs to be done by her general practitioner. The patient says: “I just mentioned it, because I realized this only when I was filling in the questionnaire, and it made me wonder if my blood pressure was too high. I’m a bit worried and hoped that you could check it”. The physician ignores the patient’s request and talks no more about blood pressure or about the questionnaire, but instead asks the patient if she has their phone number and offers her a card with contact information. The patient seems a bit disappointed that the physician did not take action on the problem.* (Participant observation April 2017).

These signs were retrieved from the individual patient and clinician interviews. Filling in the PRO questionnaire raised patients’ expectations regarding the PRO consultation. First of all, they expected that their responses would be applied in the consultation and that the clinicians knew their answers. If the clinician seemed unprepared, the patient sometimes felt that it had been useless to fill in the questionnaire. They also expected that problems identified in their answers would inform the dialogue with the clinicians. Given that the questionnaire was provided and used by the epilepsy outpatient clinic, the patients expected that clinicians would be interested in all the problems mentioned in the questionnaire. In addition, several patients expected that the clinicians would provide some sort of solution to their problems. Demanding action, they found it dissatisfactory if the clinicians only noted their problems, but failed to react to them. As a 57-year-old man put it:

*“It’s simply not enough just to register that there is a problem. They simply have to act on it too, or it makes no sense to fill it in. If I register a problem, then I expect that they can help me with it somehow; otherwise why would they ask?”*

Patients acknowledged that the outpatient clinicians might not be able to handle all their problems. Nevertheless, they expected that if this was the case, the clinicians as a minimum would refer them to other relevant professionals. When these expectations were not met, the patients found it a waste of time to fill in the questionnaire, and they felt foolish and described the dialogue with the clinician as superficial. Often, patients found it incomprehensible when the clinician advised them to discuss the problem with their general practitioner.

The clinicians also recognized that PRO-based follow-up gave rise to certain patient expectations regarding the PRO consultations. They emphasized that a patient would often expect them to solve the problems noted in the PRO questionnaire. If the problem was not related to the medical treatment, the clinician often found that they had no problem-solving options. Thus, they emphasized that PRO-based follow-up could unearth a load of unrealistic expectations, which they perceived as deeply unsatisfactory. A physician explained:

*“Because they do provide answers to some things, but how’s that related to epilepsy? And what I sometimes feel is that people expect you to explain everything to them, and then simply get started providing a solution or a treatment. And that’s miles away from what I can do. And what I’ve experienced is that the patient was disappointed because we’d talked about sex life and he wanted a solution and this and that, and then I said that this demands a though work up, and treatment is not simply about handing out pills and such. They reckon that when I ask a question, then I’ll also be taking action in relation to it.”*

Some clinicians experienced that PRO-based follow-up resulted in feelings of professional inadequacy when they were incapable of accommodating the patients’ expectations.

## Discussion

We found much variation in the degree to which PRO measures were applied and articulated in consultations. Deliberate use of PRO measures can make a positive difference for patients’ experiences; patients found that PRO measures made consultations more relevant and personal. Overall, they also found that PRO measures improved communication with the clinician. Clinicians also recognized a positive effect, reporting that PRO measures supported the patients’ active engagement in the consultation and improved communication. Several of our findings confirm extant literature on the use of PRO measures in clinical practice: specifically, the ability of PRO measures to improve communication [16, 17, 19, 29], the variable degree to which PRO measures were applied and articulated in the consultations, and the patients’ experiences of the difference importance assigned to PRO measures by clinicians [22, 29, 30]. Thus, our findings are in line with those of a study of how doctors refer to PRO measures in oncology consultations [22]. This study documented that PRO measures were often not explicitly referred to by either doctors or patients, and that high scores on the PRO data were not explored further if the patient indicated that they were not a problem or were unrelated to the current disease or treatment [22].

We found some mismatch between patients’ expectations regarding how their PRO data should be addressed and acted on and the way clinicians addressed and acted on these measures. Unfulfilled expectations negatively influenced patients’ overall experience of follow-up. A notion of unrealistic expectations as a result of PRO measures is documented in a qualitative study of patient and clinician perspectives on electronic PRO (ePRO) measures in advanced kidney disease [31]. This study found that clinicians were concerned that an ePRO system might raise patient expectations to unrealistic levels; however this study retrieved no such unrealistic expectations among the patient participants. To our knowledge, our study is the first to document such expectations in relation to the use of PRO measures among patients. Perhaps, one way to better adapt patients’ expectations to the clinical situation would be to train clinicians in deliberate use of PRO measures in the patient-clinician interaction. Such a notion would be in line with research in oncology, pediatrics, and lung transplant patients, which argues that there is a need for teaching clinicians how to use PRO measures and for recommendations on how to respond to issues identified by PRO measures [32, 33]. It has been proposed that clinicians’ lack of knowledge about how to effectively utilize and respond to PRO measures limits their successful implementation in clinical practice [32, 33]. However, as responding to symptoms is a core focus in clinical training that may outrank attending to functioning and well-being, it may, indeed, be difficult for clinicians to respond adequately to all issues identified by PRO measures [32]. Our findings seem to support this view as clinicians reacted mainly to PRO measures related to seizure or pharmacologic treatment. It has also been suggested that PRO measures could be brought to better use in routine care if clinicians receive guidance on how to address the issues identified. Such guidelines have been developed for oncology [32]. They emphasize the need to assess and evaluate the history and nature of the specific issues identified by PRO measures. They also suggest that if a patient problem is discovered, a range of responses should be considered, from pharmacologic treatment to lifestyle modifications and referral to other experts in a multidisciplinary team. A range of similar responses to the PRO measures in AmbuFlex/epilepsy could help better meet patients’ expectations.

A strength of our study is the multi-perspective design. Participant observations provided us with a platform to reflect upon the behavior of patients and clinicians in relation to PRO measures that was not overly influence by patients’ and clinicians’ subjectivity. Combining the observations with both informal interviews with clinicians afterwards and semi-structured interviews with some of the patients and clinicians, we gained access to their perspectives on what we observed and thereby a more nuanced perspective on PRO measures’ influence on the patient-clinician interaction.

There are some limitations to our study. We are aware of the potential risk of having interviewed patients willing to share their experiences because they had mainly positive experiences. However, the empirical material revealed broad and nuanced patient perspectives on how PRO measures affected there interaction with the clinician. Furthermore, all participants seemed willing to share both disappointing and positive experiences of the follow-up. Another limitation is that the participant observations included only two nurse PRO consultations. We found indications that nurses applied PRO measures in a more structured and thorough manner than physicians. More observations of nurse consultations could have helped us further explore this potential difference between the professions. It might also have been beneficial to include empirical material based on the nurses’ telephone conversations with patients as we assume that these conversations could have discussed the patient’s PRO measures to a greater extent than the physical PRO consultations. Thus, further research should include more observations relating to nurse consultations and include telephone as well as physical encounters.

Concerning transferability, we consider that although patients with epilepsy are used as an exemplar case in our study, the results are relevant in other clinical contexts where PRO measures are applied in the routine follow-up for other long-term conditions.

## Conclusion

Applying and articulating PRO measures in the consultation affects patient-clinician interaction. PRO measures can guide consultations as patients’ PRO data may inform a dialogue on psychosocial problems, among other issues. Furthermore, PRO measures may make patient-clinician communication more patient-centered as they prompt patients to express emotions and disclose fears and worries. However, clinical initiatives were taken mainly in response to PRO measures related to seizures or pharmacologic treatment. In addition, PRO-based follow-up may give patients expectations regarding the encounter. Often these expectations are not meet, which can give patients a negative experience of the interaction and the follow-up.

### Practice implications

We stress the importance of the application and deliberate use of patients’ PRO measures to enhance patient-clinician interaction and make patients more involved in clinical decision-making. We suggest that clinicians receive ongoing training in deliberate use of PRO measures in the patient-clinician interaction. Summarizing and reporting the patient’s PRO data back to the patient and the clinician would probably enhance the value of the PRO measures even further as the patient would be better prepared for consultations and able to participate more. We also propose that careful consideration should be given to which specific PRO measures to include, as there seems to be a mismatch between the PRO measures selected and what the physicians can address during the PRO consultation. Finally, we recommend that attention be paid to patients’ introduction to PRO-based follow-up in order to clarify expectations. Health care professionals need to take the patients’ expectations regarding PRO measures into account and clarify what they can offer in this respect.

## Supplementary information

**Additional file 1.** The AmbuFlex/epilepsy questionnaire.

## Data Availability

Not applicable.

## Abbreviations

ID: Interpretive description
PRO: Patient-reported outcome
